# Coronary artery wall contrast enhancement imaging impact on disease activity assessment in IgG4-RD: *a direct marker of coronary involvement*

**DOI:** 10.1016/j.jocmr.2024.101047

**Published:** 2024-05-31

**Authors:** Yaqi Du, Shuang Ding, Ce Li, Yun Bai, Xinrui Wang, Debiao Li, Yibin Xie, Guoguang Fan, Lian-Ming Wu, Guan Wang

**Affiliations:** a Department of Radiology, the First Hospital of China Medical University, Shenyang, China; b Department of rheumatology and immunology, the First Hospital of China Medical University, Shenyang, China; c Department of Medical Oncology, the First Hospital of China Medical University, Shenyang, China; d Biomedical Imaging Research Institute, Cedars Sinai Medical Center, Los Angeles, California; e Department of Radiology, Renji Hospital, School of Medicine, Shanghai Jiao Tong University, Shanghai, China

**Keywords:** IgG4-related cardiovascular disease, Cardiovascular magnetic resonance, Coronary wall contrast enhancement, IgG4-RD responder index

## Abstract

**Background:**

Coronary artery wall contrast enhancement (CE) has been applied to non-invasive visualization of changes to the coronary artery wall in systemic lupus erythematosus (SLE). This study investigated the feasibility of quantifying CE to detect coronary involvement in IgG4-related disease (IgG4-RD), as well as the influence on disease activity assessment.

**Methods:**

A total of 93 subjects (31 IgG4-RD; 29 SLE; 33 controls) were recruited in the study. Coronary artery wall imaging was performed in a 3.0 T MRI scanner. Serological markers and IgG4-RD Responder Index (IgG4-RD-RI) scores were collected for correlation analysis.

**Results:**

Coronary wall CE was observed in 29 (94 %) IgG4-RD patients and 22 (76 %) SLE patients. Contrast-to-noise ratio (CNR) and total CE area were significantly higher in patient groups compared to controls (CNR: 6.1 ± 2.7 [IgG4-RD] *v*. 4.2 ± 2.3 [SLE] *v*. 1.9 ± 1.5 [control], *P* < 0.001; Total CE area: 3.0 [3.0–6.6] *v*. 1.7 [1.5–2.6] *v*. 0.3 [0.3–0.9], *P* < 0.001). In the IgG4-RD group, CNR and total CE area were correlated with the RI (CNR: *r* = 0.55, *P* = 0.002; total CE area: *r* = 0.39, *P* = 0.031). RI´ scored considering coronary involvement by CE, differed significantly from RI scored without consideration of CE (RI *v*. RI´: 15 ± 6 *v*. 16 ± 6, *P* < 0.001).

**Conclusions:**

Visualization and quantification of CMR coronary CE by CNR and total CE area could be utilized to detect subclinical and clinical coronary wall involvement, which is prevalent in IgG4-RD. The potential inclusion of small and medium-sized vessel involvements in the assessment of disease activity in IgG4-RD is worthy of further investigation.

## Graphical Abstract

**Figure:**
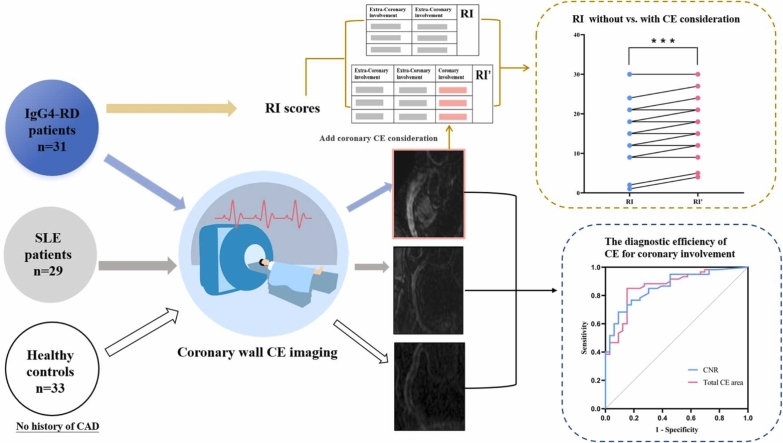
Both CE quantification measures have good diagnostic efficiency to discriminate between control subjects and patients. In the IgG4-RD group, RI´ scored considering coronary involvement by CE, differed significantly from RI scored without. IgG4-RD: IgG4-related disease; SLE: Systemic lupus erythematosus; CAD: Coronary artery diseases; RI: Responder Index; CE: Contrast enhancement; CNR: Contrast to noise ratio.

## 1 Background

IgG4-related disease (IgG4-RD) is a rare systemic fibro-inflammatory disorder characterized by the presence of tumor-like masses that can affect multiple organs. In IgG4-related cardiovascular disease (IgG4-RCVD), common sites of involvement include the large and medium arteries (aorta, pulmonary artery, peripheral arteries), coronary arteries, pericardium, myocardium and heart valves.[1] Coronary arteries are the second most commonly affected extra-aortal arteries in IgG4-RD with periarteritis.[2] There have been reports of sudden death in IgG4-RD patients attributed to coronary involvement, even in cases without peri-coronary soft tissue masses being present.[3], [4].

Due to the lack of significant clinical symptoms and specific laboratory markers, early detection of coronary involvement in patients with IgG4-RD is challenging and relies heavily on imaging evidence. Computed tomography angiography (CTA) imaging has shown the ability to identify coronary stenosis, tumor-like lesions, coronary ectasia, and aneurysm formation.[5] These CTA findings often indicate advanced stages of coronary inflammation, by which assessment of coronary involvement may underestimate disease extent and progression. It is crucial to identify coronary wall involvement in subjects with persistent systemic inflammation in the early stages to prevent adverse events. [6], [7] Previous studies have confirmed the feasibility of coronary artery wall contrast enhancement (CE) using cardiovascular magnetic resonance (CMR) to detect coronary vasculitis in systemic lupus erythematosus (SLE).[8] Nevertheless, it remains uncertain whether the introduction of coronary artery wall CE imaging is feasible for detecting early subclinical coronary vessel wall involvement in patients with IgG4-RD.

In addition, the IgG4-Related Disease Responder Index (IgG4-RD RI) has been established as a reliable instrument for evaluating disease activity and recording disease-associated damage in the same or different organs.[9] Changes in the RI scores reflect disease exacerbation or improvement, indicating the requirement for increased or decreased treatment, respectively.[9] However, there are currently no studies specifically investigating the use of the RI to evaluate cardiovascular involvement. It remains uncertain as to whether the introduction of coronary wall imaging would influence the interpretation of disease activity based on the IgG4-RD RI.

This study aims to assess the feasibility of using coronary wall CE quantification parameters, including contrast-to-noise ratio (CNR) and total CE area, for detecting clinical and subclinical coronary wall involvement in patients with IgG4-RD. The potential influence of introducing coronary CE on the assessment of disease activity was also investigated.

## 2 Methods

An observational study was undertaken in our center from April 2021 to February 2023. We recruited 33 consecutive subjects diagnosed with IgG4-RD according to the 2019 American College of Rheumatology (ACR)/European League Against Rheumatism (EULAR) classification criteria (Detailed clinical diagnostic criteria please refer to the additional file).[10] Additionally, 30 patients diagnosed with SLE out of the 41 recruited during the same period were randomly included based on the 2019 EULAR/ACR classification criteria. [11] None of the subjects had a history of coronary artery disease (CAD). During the same period, we also recruited 33 volunteers served as the control group, these individuals with neither cardiac symptoms nor regular use of medication, were willing to undergo contrast-enhanced coronary CTA as part of their health assessment and had normal CTA, echocardiography and CMR findings. Patient characteristics, including age, sex, cardiac function and symptoms, presence of cardiovascular risk factors, and medication, were recorded for all subjects. The study protocol was reviewed and approved by the ethics committee of The First Affiliated Hospital of China Medical University. Written informed consent was obtained from all participants.

In all IgG4-RD patients, serological markers were measured, including IgG, IgG4, C3, C4, IgE, eosinophils, erythrocyte sedimentation rate (ESR), and C-reactive protein (CRP). The disease activity of IgG4-RD was evaluated using the IgG4-RD RI, which included a category for cardiac involvement labelled “heart/pericardium”. Within this category, abnormalities that were related to myocardial, cardiac function, pericardial and coronary involvement were considered. The RI scores from various organ systems were combined, and the final score for the “heart/pericardium” category did not exceed 3 points (Detailed scoring methods refer to additional file). [9].

### 2.1 CMR image acquisition and analysis

All patients underwent CMR imaging using a 3.0 T clinical TX multi-source emission magnetic resonance instrument (Magnetom Verio, Siemens Healthcare, Germany) [gradient strength, 80 mT/m, gradient switching rate, 200 T/m/s and 32 channel surface phased array coil with ECG respiratory gating board were used. The acquired sequences and parameters are as follows:

Steady state free precession (SSFP) cine sequence: TR, 51.5 ms; TE, 1.7 ms; flip angle (FA), 70°; field-of-view (FOV), 340 × 360 mm2; acquisition matrix 256 × 192; short-axis SSFP cine sequence scanning thickness of 8 mm; scanning range from ventricular base to apex. Slice-matched short-axis native T1, T2 maps, and post-contrast T1 maps covering the entire left ventricle were acquired. T1map was generated by MOLLI sequence: 8 inversion time [TI], 2 Look-Locker cycles for 3 + 5 images, minimum TI= 120 ms, TI increment = 80 ms, Flip angle = 35°, read out band width = 1002 Hz/ pixel. Late gadolinium enhancement (LGE) imaging was conducted approximately 15 min after intravenous administration of gadopentetate dimeglumine (0.2 mmol/kg of body weight, Bayer, Germany), and was performed by phase sensitive inversion recovery (PSIR) with ECG gated breath hold: TR, 750 ms; TE, 2.6 ms; inversion time (TI) was usually 250–320 ms; FOV, 340 × 360 mm2; acquisition matrix 256 × 192; scanning range was the same as cine sequence; scanning slice thickness was 2 mm.

All acquired images were transferred to CVI42 software (Version 5.6.3, Circle Cardiovascular Imaging, Calgary, Alberta, Canada) for post-processing and analysis. Endocardial left ventricle (LV) borders were automatically traced at end-diastole end-systole. The papillary muscles were included as part of the LV cavity volume. Cardiac function parameters included left ventricular ejection fraction (LVEF%) and left ventricle end diastolic volume (LVEDV) index. LVEF% was computed as end-diastolic volume – (end-systolic volume/end-diastolic volume). LVEDV index was computed as EDV/body surface area (BSA). T1, T2 mapping quantification was post-processed by automatically tracing endocardial and epicardial contours, inclusion of blood pool or adjacent tissue should be carefully avoided. [12], [13] Extracellular tissue volume (ECV) was calculated as ECV%= (1-haematocrit)x[ΔT1myocardial]/[ΔT1blood]. [14].

### 2.2 Coronary wall image acquisition and analysis

The Coronary Atherosclerosis T1-Weighted Characterization with Integrated Anatomical Reference (CATCH) sequence was employed to obtain both dark-blood and bright-blood coronary images.[15] The scans were conducted while the subjects were breathing freely, and respiratory navigation was utilized. Preparatory scans were performed before the CATCH sequence including the following steps: 1) low-resolution 2-dimensional survey images with multiple orientations to localize the heart; 2) multi-echo gradient echo sequence for cardiac shimming; and 3) free-breathing 4-chamber cine images of the heart to determine the trigger delay time when the motion of the coronary arteries was minimal. After 25 min of delay time to allow contrast media to wash out, a Look-Locker type sequence was used to determine the optimal inversion time interval to null the blood pool signal. [15].

CATCH sequence was executed with the following parameters: 3-dimensional transverse slab covering the entire heart with field-of-view = 322 × 242 × 108 mm^3^; matrix size = 248 × 188 × 84; acquired spatial resolution = 1.3 × 1.3 × 1.3 mm^3^ (interpolated to 0.7 × 0.7 × 1.3 mm^3^); flip angle = 12°; repetition time/echo time = 4.6/2.3 ms; the optimal inversion time interval typically ranging from 100 ms to 300 ms, applied every other heartbeat; bandwidth = 721 Hz/pixel; acquisition time was approximately 12 min depending on heart rate.[15].

**Fig. 1:**
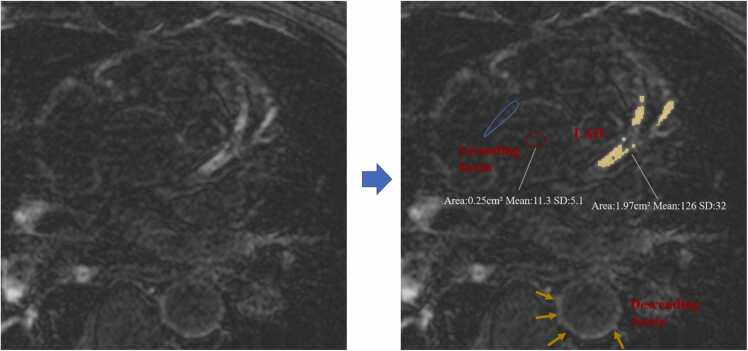
**Quantification of Coronary CE by CVI42 post-processing software.** Contrast enhancement in the LAD coronary artery wall (yellow area): SIs > 2 SDs of the non-enhancing aortic wall (blue circle). CNR: based on measuring SIs of the LAD vessel wall and blood pool (red circle). Quantitative parameter values at this slice: Total CE area= 1.97 cm². CNR= (SIwall-SIblood)/ mean (SD of both ROIs)= (126–11.3)/((32 +5.1)/2)= 6.2. Yellow arrows indicate the thickened and enhanced descending aorta wall. *CE* contrast enhancement, *LAD* left anterior descending coronary artery, *SIs* signal intensities, *CNR* contrast to noise ratio.

All images were post-processed using CVI42 software. Two independent observers, blinded to the CTA results and subject type, analyzed the data. Visual assessment was performed to evaluate the distribution of CE.[16] Coronary wall contrast enhancement (CE) was defined as areas in the vessel wall with signal intensities > 2 SDs of the non-enhancing and non-thickened aortic wall,[16] total CE area was then quantified by manual delineation of placing a region-of-interest (ROI) around proximal coronary arteries. Coronary wall signal intensities (SI) were assessed by placing a ROI tightly within the enhanced area within proximal segments of the vessel wall. Coronary blood SI was determined from ROI within the blood pool of ascending aorta within the same imaging slice as the coronary wall SI, to ensure that the image scaling was consistent. The coronary wall contrast-to-noise ratio (CNR) was calculated as follows: CNR= (SI wall-SI blood)/mean (SD of both ROIs).[8] **(**Fig. 1**)** The proximal to mid-segment of both coronary arteries are visualized in the vast majority of all subjects, CNR and total CE area are both obtained from the right coronary artery (RCA), left anterior descending(LAD) and left circumflex(LCX) artery.

### 2.3 Statistical analysis

Normality of distributions was assessed using the Kolmogorov-Smirnov statistic. Categorical data are expressed as percentages, and continuous variables as mean ± SD or median (interquartile range), as appropriate. Comparison of independent samples was performed using 1-way analysis of variance (ANOVA), Kruskal-Wallis or chi-squared test with Bonferroni *post-hoc* and Fisher exact tests, as appropriate for the type of the data. A paired *t*-test was conducted for self-control design. Associations were explored by simple linear and binary logistic regression analyses. Cut-off values for the discrimination between health and disease were derived using receiver-operating characteristic curve analysis using the point that maximized the trade-off between specificity and sensitivity. Interobserver reproducibility and agreement were performed using Bland-Altman methods. All tests were two-tailed, and a *p*-value of less than 0.05 was deemed significant.

## 3 Results

### 3.1 Reproducibility assessment

**Fig. 2:**
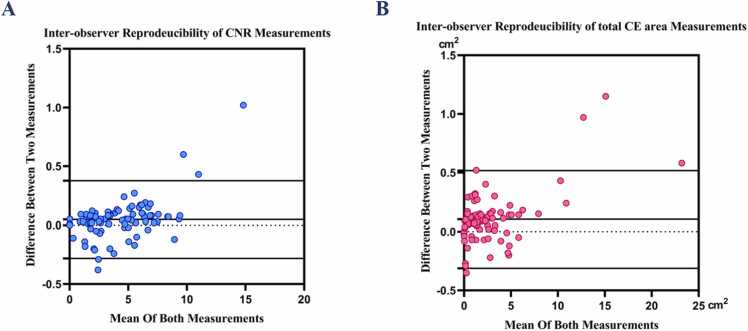
Bland Altman plots for assessment of interobserver reproducibility of CNR (A) and total CE area(B). (A). Excellent agreement in CNR quantification determined by the 2 observers: P < 0.001, MD± 2 *SD = 0.047 ± 0.336**.** (B). Good agreement in total CE area quantification determined by the 2 observers: P < 0.001, MD± 2 *SD (cm2) = 0.103 ± 0.422. *CNR* contrast to noise ratio, *CE* contrast enhancement, *MD* mean difference.

Bland-Altman analyses showed excellent agreement in CNR quantification determined by the 2 observers (P < 0.001, mean difference(MD) ± 2 *SD = 0.047 ± 0.336) **(**Fig. 2**A).** Assessment of total CE area also showed a good Inter-observer reproducibility. (P < 0.001, MD±2 *SD (cm2) = 0.103 ± 0.422). **(**Fig. 2**B)**.

### 3.2 Patient characteristics

One SLE patient and two IgG4-RD patients were excluded from the study due to severe coronary lumen stenosis and myocardial infarction detected on CMR and CTA findings. Eventually, a total of 93 subjects were enrolled in the study, including 31 patients with IgG4-RD, 29 patients with SLE, and 33 control subjects. The IgG4-RD group had a higher average age compared to the SLE group and control subjects (*P* < 0.001). There was a predominance of males in the IgG4-RD group, while all SLE patients were female. In the IgG4-RD group, three patients experienced mild to moderate cardiac symptoms such as palpitations and shortness of breath. All IgG4-RD patients demonstrated involvement of multiple organs (≥ 3), most commonly affecting the salivary glands (97 %) and lymph nodes (88 %). Pancreatic involvement was observed in nine patients (29 %). Extra-coronary cardiovascular involvement was presented in 14 patients (45 %) and primarily in the myocardium, while large vessel involvement was presented in three patients (10 %). **(**Table 2**)** Seven IgG4-RD patients had received disease-modifying agents before admission, including glucocorticoids (*n* = 7), immunosuppressive agents (*n* = 2), or a combination of both (*n* = 2), with the median duration of 455 days (Q1 90 - Q3 548). The mean RI score for all enrolled IgG4-RD patients in this study was 15. Among the SLE patients, 12 individuals developed mild to moderate cardiac symptoms, and 25 patients (86 %) exhibited extra-coronary cardiovascular involvement, mainly affecting the myocardium. Twenty-three SLE patients had received disease-modifying agents before admission, including glucocorticoids (*n* = 22), immunosuppressive agents (*n* = 15), biological agents (*n* = 7), and hydroxychloroquine (HCQ) (*n* = 15), with the median duration of 730 days (Q1 3285 - Q3 5840). The mean Systemic Lupus Erythematosus Disease Activity Index (SLEDAI) score for all enrolled SLE patients in this study was 14 **(**Table 1, Table 2**)**.

**Table 1:** Patient Characteristics.

	Controls (n = 33)	IgG4-RD (n = 31)	SLE (n = 29)	P Value
Age,yrs	53 ± 7	**60 ± 10**^**#**^	37 ± 15†	＜0.001
Male	14(42)	**16(52)**^**#**^	0(0)†	＜0.001
Heart rate, beats/min	71 ± 17	**68 ± 12**^**#**^	78 ± 14	0.026
Positive cardiac symptoms	0(0)	**3(10)**^**#**^	12(41) †	＜0.001
Hypertension	0(0)	**3(10)**	8(26) †	0.009
Diabetes	0(0)	**1(3)**	0(0)	0.130
Smoking	5(15)	**4(13)**	0(0)	0.100
Hypercholesterolemia	1(3)	**0(0)**	2(7)	0.318
Extra-cardiovascular organs involvement (mean)	-	**4**	2	
Treatment	-	**7(23)**^**#**^	23(79)	
Glucocorticoid	-	**7(100)**^**#**^	22(96)	
Immunosuppressive agents	-	**2(29)**^**#**^	15(65)	
Biological agents	-	**-**	7(30)	
HCQ	-	**-**	15(65)	
Duration, days	-	**455(90-548)**^**#**^	3285(730-5840)	
IgG4-RD RI	-	**15 ± 6**	-	
SLEDAI	-	**-**	14 ± 9	

Values are mean±SD or n (%).

One-way analysis of variance and Kruskall-Wallis with post-hoc tests for differences, P value for the One-way analysis of the whole corhort.

*for controls vs. IgG4-Related Disease (IgG4-RD) patients, †for controls vs. systemic lupus

erythematosus (SLE) and ^#^for IgG4-Related Disease (IgG4-RD) patients vs. systemic lupus

erythematosus (SLE), represent statistical significance on post-hoc tests between each two groups.

Immunosuppressive agents include Leflunomide, Mycophenolate sodium, Tripterygium glycosides, Thalidomide, Tacrolimus, Ciclosporin, Cyclophosphamide, Methotrexate. Biological agents include Telitacicept, Belimumab.

Extra-cardiovascular organs involvement include Salivary glands, Lymph nodes, Nasal cavity/sinus, Tonsil, Thyroid, Lung, Retroperitoneal fibrosis, Mediastinum, Pancreas, Liver, Bile duct, Kidney, Meninges, Pituitary gland, Orbit, Prostate glands, Pleura, Cerebrum, Skin, Arthrosis, Myositis.

*SLE* systemic lupus erythematosus, *IgG4-RD* IgG4-related disease, *HCQ* hydroxychloroquine, *RI* responder index, *SLEDAI* Systemic Lupus Erythematosus Disease Activity Index.

**Table 2:** Characteristics by Cardiac Magnetic Resonance.

	Controls (n = 33)	**IgG4-RD (n = 31)**	SLE (n = 29)	P Value
LVEF (%)	60 ± 7	**58 ± 7**	55 ± 7†	0.048
LVEDVindex, ml/m2	66 ± 12	**64 ± 16**^**#**^	76 ± 16†	0.003
LGE, n present	0(0)	**2(6)**	5(17)†	0.023
T1 Map, ms	1267 ± 68	**1270 ± 54**^**#**^	1366 ± 83†	＜0.001
T2 Map, ms	42 ± 2	**42 ± 2**^**#**^	45 ± 4†	＜0.001
ECV%	30 ± 2	**30 ± 3**	31 ± 4	0.420
Strain				
radial	40 ± 15	**40 ± 9**^**#**^	35 ± 12	0.189
circumferential	-20 ± 3	**-21 ± 2**^**#**^	-19 ± 3	0.039
longitudinal	-13 ± 3	**-15 ± 3**	-13 ± 3	0.053
Cardiovascular involvement				
myocardium	0(0)	6(19)*^#^	19(66)†	＜0.001
pericardium	0(0)	5(16)*^#^	11(38)†	＜0.001
aorta	0(0)	**1(3)**	0(0)	0.364
pulmonary artery	0(0)	**2(6)**	4(14)	0.088
coronary artery wall thickening	0(0)	10(32)*^#^	1(3)	＜0.001
coronary CE	4(12)	29(94)*	22(76)†	＜0.001
CE pattern				
patchy	4(100)	**5(17)**	4(18)	0.553
generalized	0(0)	24(83)*	18(82)†	＜0.001
Total area, cm2	0.3(0.3-0.9)	3.0(3.0-6.6)*	1.7(1.5-2.6)†	＜0.001
CNR	1.9 ± 1.5	6.1 ± 2.7 *^#^	4.2 ± 2.3†	＜0.001

Values are mean±SD or n (%).

One-way analysis of variance and Kruskall-Wallis with post-hoc tests for differences, P value for the One-way analysis of the whole corhort.

*for controls vs. IgG4-Related Disease (IgG4-RD) patients, †for controls vs. systemic lupus erythematosus (SLE) and ^#^for IgG4-Related Disease (IgG4-RD) patients vs. systemic lupus erythematosus (SLE), represent statistical significance on post-hoc tests between each two groups.

*LVEF* left ventricle ejection fraction, *LVEDV,* left ventricle end diastolic volume, *LGE* late gadolinium enhancement, *ECV,* extracellular volume, *CE* contrast enhancement, *CNR,* contrast to noise ratio, *IgG4-RD* IgG4-related disease, *SLE* systemic lupus erythematosus.

### 3.3 CMR results

Regarding cardiac function, there were no significant differences in LVEF among all three groups, and the LVEDV index was similar between IgG4-RD and control subjects; however, the LVEDV index was higher in SLE patients compared to IgG4-RD and control subjects. T1 and T2 values, which indicate tissue characteristics, were not significantly different between IgG4-RD and control subjects (IgG4-RD *v*. control: T1 = 1270 ± 54 *v*. 1267 ± 68 ms, *P* = 0.99, T2 = 42 ± 2 *v*. 42 ± 2 ms, *P* = 1.000). In contrast, the SLE group had higher T1 and T2 values (T1 = 1366 ± 83 ms, T2 = 45 ± 4 ms) compared to both IgG4-RD and control subjects (*P* < 0.001) **(**Table 2**)**.

**Fig. 3:**
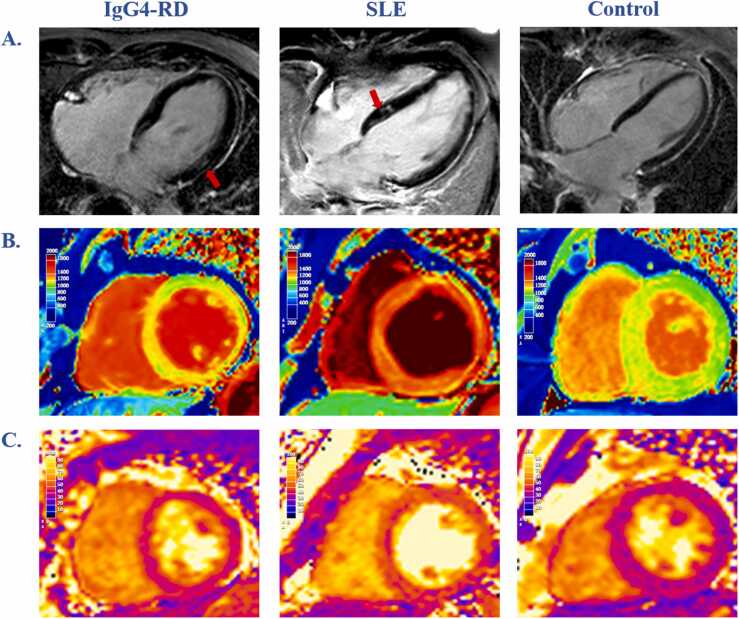
**Representative LGE, T1 and T2 map images of myocardial involvement in IgG4-RD and SLE patients.** Patchy LGE was observed in the basal mid-lateral wall of the LV in an IgG4-RD patient and the basal mid-septal wall of the LV in a SLE patient (A, red arrows). A SLE patient showed significantly higher T1 and T2 values of LV to control subject, while T1 and T2 values were mild increased in an IgG4-RD patient (B, C). (IgG4-RD *v*. SLE *v*. control: T1 = 1218 ± 84 *v*. 1413 ± 151 *v*. 1122 ± 92 ms, T2 = 44 ± 6 *v*. 53 ± 9 *v*. 39 ± 5 ms). *LGE* late gadolinium enhancement, *IgG4-RD* IgG4-related disease, *SLE* systemic lupus erythematosus, *LV* left ventricle.

Two IgG4-RD patients (6 %) showed focal myocardial fibrosis characterized by increased values of T1 and ECV%, along with LGE in the middle layer of myocardium. Additionally, four patients (13 %) were suspected of myocardial oedema characterized by mildly diffuse T2 and/or T1 elevation, one of whom had concomitant chest pain. Similar CMR manifestations of focal myocardial fibrosis in five SLE patients (17 %) and myocardial oedema in fifteen SLE patients (52 %) were observed **(**Fig. 3**)**.

In the IgG4-RD group, three patients (9 %) exhibited large vessel involvement with wall thickening and enhancement, including one involving the aorta and two involving the pulmonary artery. Pericardial involvement could be found in five patients (16 %), with one showing pericardial enhancement and the remaining four showing pericardial effusion **(**Table 2**)**.

### 3.4 Coronary CE image analysis

The proximal coronary arteries (proximal to mid-segment) were evaluated in all subjects and included in the visual assessment and quantification analysis of coronary CE. The image quality was judged using a modified 4-point Likert scale as following, with a mean score of 3.2:

1. Poor (*n* = 2): impaired image quality, the proximal to mid-segment of two of the main coronary arteries was visible and the lumen and wall signals could be identified with reduced confidence.
2. Moderate (*n* = 4): reduced image quality because of acceptable artifacts, the proximal to mid-segment of two of the three main coronary arteries were clearly visible and most of the lumen and wall signals could be identified.
3. Good (*n* = 57): slightly reduced image quality because of few artifacts, the proximal to mid-segment of the three main coronary arteries were clearly visible and most of the lumen and wall signals could be clearly distinguished.
4. Excellent (*n* = 30): no artifacts present, the proximal to mid-segment of the three main coronary arteries were clearly visible and all the lumen and wall signals could be clearly distinguished.

Using binary logistic regression and receiver-operating characteristic analysis, the high agreements were observed between CE quantification measures and clinical diagnosis (total CE area [cut-off: 0.99 cm^2^]: sensitivity 85 %, specificity 85 %, area under the curve: 0.87, odds ratio = 3.65, 95 % confidence interval: 1.96 to 6.78, *p* < 0.001; CNR[cut-off: 3.85]: sensitivity 68 %, specificity 91 %, area under the curve: 0.86, odds ratio = 2.14, 95 % confidence interval: 1.55 to 2.96, *p* < 0.001) **(**Additional Fig. 1A**)**. According to the cut-off value in receiver-operating characteristic curve analysis, coronary artery with a total CE area greater than, or equal to 0.99 cm² with or without morphological changes was considered positive for coronary CE, whereas coronary artery with a total CE area of less than 0.99 cm² was considered negative.

**Fig. 4:**
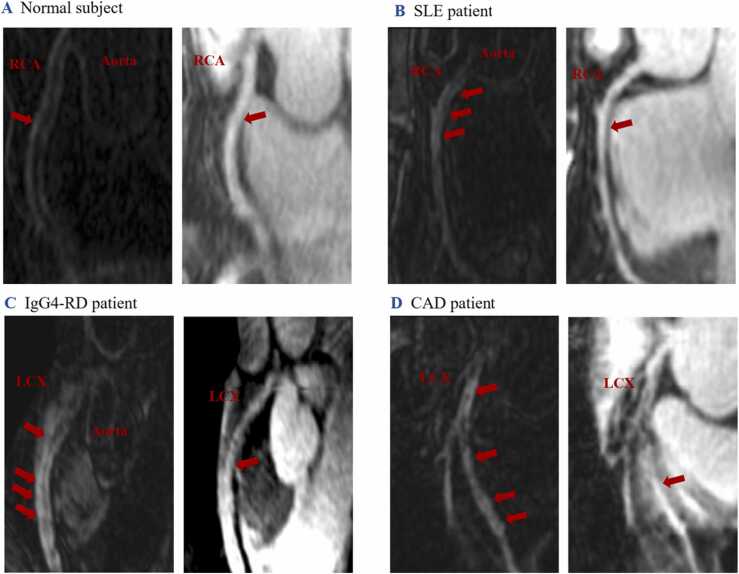
**Representative coronary images in normal control subject, SLE patient, IgG4-RD patient and CAD patient.** Coronary CE and wall thickening were observed in both IgG4-RD (C) and SLE patient (B) with a diffuse pattern without luminal stenosis while control subject manifested as mild patchy coronary CE (A). CAD patient showed patchy and diffuse coronary CE with severe luminal stenosis. (D). *IgG4-RD* IgG4-related disease, *SLE* systemic lupus erythematosus, *CAD* coronary artery disease, *CE* contrast enhancement, *RCA* right coronary artery, *LCX* left circumflex coronary artery.

Both patient groups had higher CNR and total CE area than the control group (*P* < 0.001). The IgG4-RD group had higher CNR than the SLE group, while there was no significant difference in the total CE area between the two patient groups (CNR: *P* < 0.001; the total CE area: *P* = 0.077). Sporadic coronary CE was observed in four control subjects (13 %) with a patchy pattern, while coronary CE was prevalent in both the IgG4-RD patient group (94 %) and the SLE patient group (76 %), with a predominately diffuse pattern. **(**Fig. 4 **A, B, and C)** Ten (32 %) of the IgG4-RD patients showed visual wall thickening. Only one (3 %) SLE patient exhibited mild wall thickening.

In IgG4-RD group, only two patients did not have coronary CE, and they also did not exhibit other cardiovascular abnormalities. Almost half of the patients with CE (48 %) had other cardiovascular involvement. And the IgG4-RD patients with visual coronary-wall thickening were more likely to have additional cardiovascular involvement, particularly myocardial (40 %). **(**Table 3**)**.

**Table 3:** The relationship between coronary abnormalities and wider cardiovascular involvement in IgG4-RD patients.

	Myocardium	Pericardium	Aorta	Pulmonary artery	Non
Coronary CE (n = 29)	6(21)	5(17)	1(3)	2(7)	15(52)*
Coronary wall thickening (n = 10)	4(40)*	2(20)	1(10)	1(10)	2(20)

*CE* contrast enhancement.

* means p < 0.05 by chi-square test.

### 3.5 Correlation analysis

CNR was correlated with total CE area in the cohort as a whole and in separate groups (control: *r* = 0.83, *P* < 0.001; IgG4-RD: *r* = 0.82, *P* < 0.001; SLE: *r* = 0.87, *P* < 0.001). The total CE area and CNR of IgG4-RD patients were both positively correlated with RI scores (the total CE area: *r* = 0.39, *P* = 0.031; CNR: *r* = 0.55, *P* = 0.002). **(**Additional Fig. 2**)** After including coronary wall CE results, significant differences were found between the updated RI scores (RI´) with consideration of coronary wall CE and the prior RI scores without consideration of coronary wall CE (RI *v*. RI´ = 15 ± 6 *v*. 16 ± 6, *P* < 0.001) **(**Table 4**)** (please refer to Additional Table 1 for detailed scores).

**Table 4:** The updated response index (denoted as RI’) with consideration of coronary wall contrast enhancement versus the prior RI without consideration of coronary wall contrast enhancement.

	RI	RI’	P value
Cardiac	1 ± 1	3 ± 1	＜0.001
Non-cardiac	14 ± 5	14 ± 5	-
Total	15 ± 6	16 ± 6	＜0.001

Detailed laboratory parameters are shown in Table 5. Both coronary CE parameters in IgG4-RD patients were correlated with the serum IgG level (total CE area: *r* = 0.39, *P* = 0.032; CNR: *r* = 0.54, *P* = 0.002), while the serum IgG4 level was only correlated with the total CE area (*r* = 0.42, *P* = 0.019). Among other laboratory indicators of disease activity, the total CE area was correlated with C3 (*r* = −0.43, *P* = 0.018) and ESR (*r* = 0.45, *P* = 0.013), while CNR was only correlated with C3 (*r* = −0.49, *P* = 0.006). **(**Table 6**)**.

**Table 5:** Blood marker values of IgG4-RD patients.

Blood makers (normal values)	N	Abnormal	Blood maker value
IgG, mg/dl(860-1740)	30	15(50)	1763(1689-2505)
IgG4, mg/dl(3-201)	31	29(94)	1030(1029-2007)
C3, g/l(0.7-1.4)	30	5(17)	0.88(0.82-0.99)
C4, g/l(1.0-1.4)	30	30(100)	0.17(0.12-0.20)
ESR, mm/h(0-15)	30	16(53)	17.5(15.4-34.4)
CRP, mg/l(0-6)	30	1(3)	2.2(0.7-7.3)
IgE, IU/ml(0-100)	20	18(90)	712(618-1401)
Eosinophils, %(0.4-0.8)	31	1(3)	0.10(0.08-0.27)

Values are n or n (%) or median (interquartile range).

*CRP* C-reactive protein, *ESR* erythrocyte sedimentation rate.

**Table 6:** Results of correlation analyses in IgG4-RD patients.

	Total CE area		CNR	
	r	p Value	r	p Value
Total CE area	0.82	＜0.001		
CNR	0.82	＜0.001		
IgG	0.39	0.032	0.54	0.002
IgG4	0.42	0.019	0.28	0.126
C3	-0.43	0.018	-0.49	0.006
C4	-0.26	0.159	-0.31	0.097
CRP	0.22	0.250	-0.15	0.935
ESR	0.45	0.013	0.33	0.072
IgG4-RD RI	0.39	0.031	0.60	＜0.001

Correlations were performed using Pearson or Spearman tests, as appropriate for the type of the data.

*IgG4-RD RI* IgG4-related disease responder index, *CRP* C-reactive protein, *ESR* erythrocyte sedimentation rate.

In addition, there were good correlations between the two CE parameters and the number of the sites of cardiovascular involvement (number of involved and total CE area: r = 0.45, *P* = 0.01; number of involved and CNR: r = 0.54, *P* = 0.002).

### 3.6 Impact of disease-modifying therapy on coronary CE and RI in IgG4-RD

The statistical results, after excluding the 7 treated IgG4-RD patients, are available in the Additional File, indicating no qualitative or negative impact on the significance of the relevant statistical analysis when comparing the results prior and post their exclusion. Additionally, the correlation analysis of both CE area and CNR showed a partial improvement (r value of total CE area prior vs. post exclusion: CE area and IgG=0.39 vs. 0.58, CE area and IgG4 =0.42 vs. 0.61, CE area and ESR=0.45 vs. 0.56, CE area and RI= 0.39 vs. 0.49; r value of CNR prior vs. post exclusion: CNR and IgG4 =0.28 vs. 0.42, CNR and RI= 0.60 vs. 0.73) **(**Additional Table 3**)**, and a significant difference can still be observed between RI and RI´ scores after the exclusion of 7 treated patients (RI vs. RI´ = 16 ± 5 vs. 18 ± 5, P < 0.001).

## 4 Discussion

An insufficiently sensitive or specific imaging modality might lead to incorrect IgG4-RD RI scores regarding disease activity, especially in patients without significant clinical manifestations.[17] CE of coronary artery wall on MR images, as a novel non-invasive and radiation-free imaging marker, helps to discern quiescent lesions in the coronary artery. Our data suggest that visualization and quantification of coronary CE may provide a potential new marker of IgG4-RD coronary wall remodeling and offer a new tool for more accurate and comprehensive assessment of the disease condition.

CTA imaging could be used to recognize coronary stenosis or ectasia of lumen, and tumor-like lesion formation of vessel walls.[5] These manifestations were not commonly observed in clinical practice and may indicate that the coronary inflammation has reached its advanced stage. It is noteworthy that there have been reports of sudden death in IgG4-RD patients attributed to coronary involvement, even in cases where there were no peri-coronary soft tissue masses.[3], [4] Assessing coronary involvement using CTA alone would underestimate disease extent and progression. Detecting coronary wall remodeling in the early stage is necessary to avoid the occurrence of adverse events. The present gold standard observation method of the coronary wall such as intravascular ultrasonography (IVUS) is invasive.[18] Previous studies established the feasibility of coronary vessel wall CE for detecting coronary vasculitis in autoimmune inflammatory disease (SLE).[8] Coronary Atherosclerosis T1-Weighed Characterization with Integrated Anatomical Reference (CATCH), introduced by Xie *et al*. in 2017, is a more efficient method for assessing the coronary wall and lumen together.[15].

**Fig. 5:**
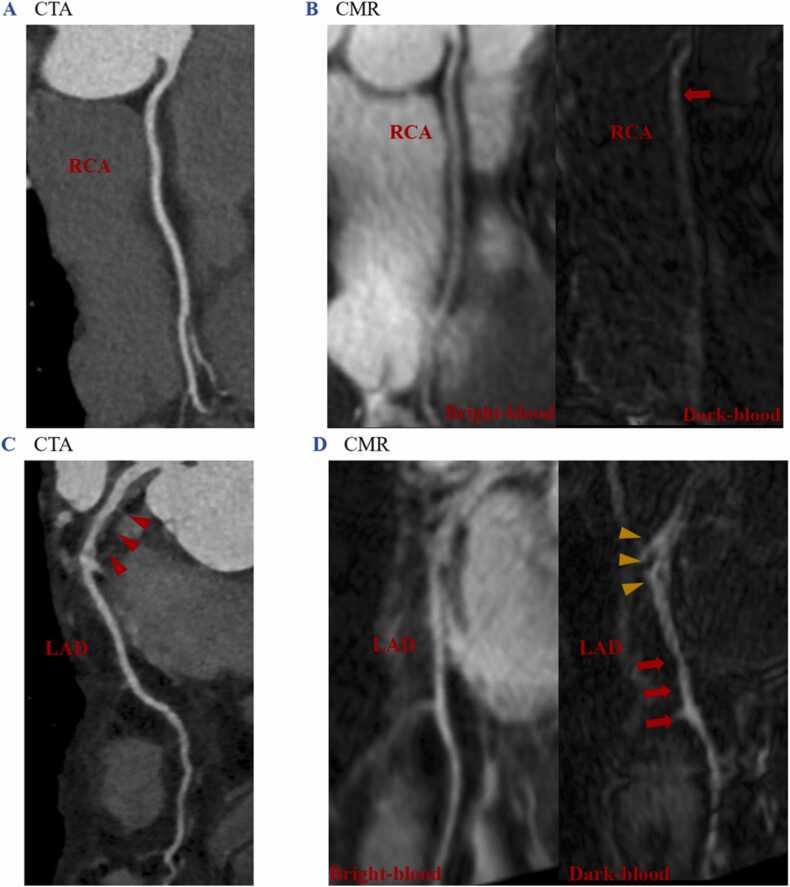
**CTA and CMR images of IgG4-RD patients with coronary involvement.** A 56-year-old female IgG4-RD patient without any cardiovascular symptoms, the CTA showed no abnormal changes (A). CMR bright-blood image showed similar ability to manifest the anatomical structure of the coronary lumen as CTA, but the accordingly dark-blood image could manifest CE in the proximal RCA wall (red arrow), suggesting coronary involvement (B). A 50-year-old male patient with IgG4-RD experienced occasional palpitations and shortness of breath. CTA indicated mild-to-moderate thickening of the proximal LAD wall (red arrowheads) (C). However, the CMR dark-blood image revealed diffuse CE in the LAD wall, involving both thickened (yellow arrowheads) and unthickened (red arrows) segments (D). *IgG4-RD* IgG4-related disease, *CTA* computed tomography angiography, *CMR* cardiovascular magnetic resonance, *RCA* right coronary artery, *LAD* left anterior descending.

In numerous studies, large vessel involvement in IgG4-RD has been extensively documented. Examples include periaortitis/periarteritis, where wall thickening devoid of calcifications is a common manifestation, marked by the engagement of the adventitia [19]. IgG4-RD coronary arterial wall involvement predominantly affects the adventitia as well [1], [20]. In contrast, SLE often exhibits involvement of the endarterium. [21] In our study, coronary CE was prevalent in both IgG4-RD and SLE patients, characterized by a predominantly diffuse pattern. Notably, no significant luminal stenosis was observed in either SLE or IgG4-RD patients, even in cases of substantial wall thickening. This distinctive feature sets these patients apart from CAD.**(**Fig. 4**D)** Ten (32 %) IgG4-RD patients in our cohort showed visual wall changes both in CMR coronary wall imaging and CTA, with only two exhibiting typical manifestations (such as tumor-like thickening of the coronary wall and aneurysmal dilatation) with neither luminal stenosis nor myocardial ischemia. We found that coronary CE is substantial (visually and quantifiably), mainly presenting a diffuse CE pattern in patients with IgG4-RD and SLE, whereas control subjects showed relatively little contrast between uptake and lower CNR values. Our results suggested qualitative assessment of coronary CE allows for the discrimination between health and diseases: coronary CE can be adopted as a new imaging marker for the detection of potential coronary wall involvement. CE of the coronary artery wall on MR images could indicate coronary wall inflammatory activities even in a subclinical stage, providing a more detailed characterization of vessel wall pathologies than conventional imaging approaches such as CTA. **(**Fig. 5**)** In addition, in IgG4-RD group, coronary CE occurred not only in patients with other cardiovascular abnormalities, but also in patients without. Good correlations were shown between the two CE parameters (total CE area and CNR) and the number of sites of cardiovascular involvement. And IgG4-RD patients with visual coronary wall thickening were more likely to have additional cardiovascular involvement. It further highlights the excellent ability of coronary CE to identify both clinical and subclinical coronary involvement in IgG4-RD. Furthermore, the wall thickening in IgG4-RD was more significant than that in SLE (as confirmed both visually and quantitatively). The total CE area and CNR in IgG4-RD patients were both higher than those in SLE patients. These findings may be attributed, in part, to the fact that a significantly higher percentage of SLE patients (79 %) had received disease-modifying agents before admission compared to the IgG4-RD group (23 %). Exploring the differences in coronary involvement between these two patient groups warrants future investigation in a cohort with more tightly controlled variables. It is important to note that this aspect is beyond the scope of our current study focus.

Measurement of disease activity is critical for longitudinal assessments in observational studies and clinical trials of IgG4-RD. Disease activity reflects a patient’s symptoms attributable to active IgG4-RD as well as significant findings from the physical examination, imaging studies, and laboratory evaluations. Serum IgG4, C3/C4, CRP and ESR can be used as laboratory markers of disease activity for clinical application.[22] Many investigators found that patients with vascular involvement of IgG4-RD have higher levels of serological inflammatory markers than those without.[20] The total CE area was significantly associated with serum IgG4 and ESR, while only one of all IgG4-RD patients showed increased CRP. Previous studies have found that the serum IgG4 level elevated in 60–70 % of active untreated patients,[23] while CRP and ESR are markers of inflammation with relatively low specificity.[24], [25] It is widely acknowledged that the ESR is typically elevated in active SLE patients, while CRP is more commonly utilized as a marker for severe infections.[26] Additionally, due to the more rapid decline of CRP levels compared to ESR during inflammation resolution, ESR is deemed more advantageous for monitoring chronic inflammation, including in cases of IgG4-RD.Therefore, these considerations may similarly account for the preference of ESR over CRP in our study. However, CNR was associated with neither serum IgG4, CRP, nor ESR. CNR represents the degree of CE, but the miscellaneous or simultaneous presence of the inflammatory states may lead to quantitative variability and instability in the overall degree of CE.

IgG4-RD RI was used to assess disease activity organ by organ, with the sum of organ assessments summed to provide a total score. The changes in the RI reflect disease flares or improvements, indicating increased or decreased treatment requirements, respectively. [9] The majority of IgG4-RD patients responded well to dose-dependent steroids treatment, even in cases with relapse.[27] A correct and timeous recognition of RI, as well as corresponding appropriate intervention, are crucial for the treatment of IgG4-RD. However, due to the hidden onset of IgG4-RCVD, especially in the early stage or in non-elderly population, investigators would have difficulty assessing disease activity in this site, relying heavily on imaging to gauge both presence and degree. In the current RI scoring schedule, coronary artery involvement was included within the category “heart/pericardium” and was not separately categorized.[9] Consequently, in some cases, the scores reached the maximum limit before considering coronary CE, resulting in no change of RI´ compared to RI scores as shown in Table 4 and Additional Table 1. This phenomenon is primarily attributed to extra-coronary cardiac involvement. However, significant differences were still observed between prior and updated RI scores when incorporating coronary CE results. It may be worth considering the inclusion of a separate scoring column for small and medium-sized vessel involvements to improve the accuracy of disease activity assessment.

To the best of our knowledge, this is the first study using visual and quantitative CMR-coronary wall imaging to recognize IgG4-RD coronary wall involvement and to apply it to the assessment of disease activity. Additionally, our study also presented the involvement of myocardium. The currently existing reported cases of myocardial involvement of IgG4-RD mostly presented as intracardiac/myocardial mass/tumor-like lesions,[28], [29] and there has been no study related to characterization of myocardial tissues. Our analysis showed that the T1 and T2 map values of myocardium in SLE patients were significantly higher than control subjects. Myocardial edema was suspected in only four IgG4-RD patients manifesting as significantly increased T2 and T1 values **(**Fig. 3**B and C)**, indicating that SLE patients were more susceptible to myocardial inflammatory infiltration. In addition, patchy LGEs in subepicardial and/or mid-layer myocardium were observed in both IgG4-RD and SLE patients, which can often represent non-ischemic myocardial injury and can be induced by inflammatory infiltration **(**Fig. 3**A)**. Whether these findings suggest IgG4-RD patients tend to have myocardial inflammatory infiltration warrants further investigation.

## 5 Limitations

This was an exploratory and hypothesis-generating pilot study with a limited sample size. The emerging coronary imaging sequence ‘CATCH’ was used to verify the feasibility of coronary CE for detecting cardiac involvement in preselected patients. Therefore, there remain certain limitations to the present study. Firstly, the study was subject to a certain patient recruitment bias because all participants were screened and matched for the assignment of patient groups. Secondly, we only focused on the proximal coronary vessels, which are adequately visualized by the CATCH technique with a high success rate. This may underlie the basis for the high reproducibility of quantitative coronary CE parameters in this study. Thirdly, quantitative measurement in the assessment of vessel wall thickening was challenging due to the small size of some vessels, thus we only adopted visualization in our evaluation, which may entail a certain subjectivity. Fourthly, data on soluble IL-2 receptor and circulating plasmablasts was not included in the study due to the incompleteness and unavailability at our center, which may have contributed to the observed lower correlation of the laboratory indicators. Finally, although the paired t-test showed that there was a significant statistical difference between the prior RI and updated RI´ scores, the difference in the mean RI scores was quite small with a large SD, which may limit the impact of adding coronary CE to the RI score. In addition, the value of CMR in monitoring the prognosis of cardiovascular involvement remains unclear (a direction not considered in the present study).

## 6 Conclusion

Visualization and quantification of CMR coronary CE by CNR and the total CE area can be utilized to detect subclinical and clinical coronary wall involvement, which is prevalent in IgG4-RD. The potential inclusion of small and medium-sized vessel involvements in the assessment of disease activity in IgG4-RD is worthy of further investigation.

## Ethics approval and consent to participate

This study involves human participants and was approved by the Ethics of Committees of The First Hospital of China Medical University. Patients gave informed consent prior to participation.

## Competing Interests

None.

## Funding

The Natural Science Foundation of Liaoning Province (2022-BS-143), The Natural Science Foundation of Shenyang City (22-321-33-31)

## CRediT authorship contribution statement

**Shuang Ding:** Supervision, Resources, Methodology, Data curation. **Yaqi Du:** Writing – original draft, Visualization, Validation, Software, Resources, Methodology, Investigation, Formal analysis, Data curation, Conceptualization. **Guan Wang:** Writing – review & editing, Visualization, Validation, Supervision, Software, Resources, Project administration, Methodology, Investigation, Formal analysis, Data curation, Conceptualization. **Lianming Wu:** Investigation. **Guoguang Fan:** Validation, Supervision, Project administration. **Yibin Xie:** Validation, Supervision, Methodology, Investigation. **Debiao Li:** Visualization, Validation, Supervision, Investigation. **Xinrui Wang:** Resources, Formal analysis, Data curation. **Yun Bai:** Resources, Formal analysis, Data curation. **Ce Li:** Supervision, Methodology, Investigation.

## Data Availability

The data underlying this article will be shared upon reasonable request from the corresponding authors.
