# Supplementary material for: Coronary artery wall contrast enhancement imaging impact on disease activity assessment in IgG4-RD: a direct marker of coronary involvement
Source: J Cardiovasc Magn Reson. 2024 May 31;26(2):101047. doi: 10.1016/j.jocmr.2024.101047 (PMC11268104; doi:10.1016/j.jocmr.2024.101047)
Supplement: Supplementary file 1 — Supplementary material. [file mmc1.docx]

**ADDITIONAL FILE**

**METHODS**

**Clinical diagnostic criteria of** **IgG4-RD (10)**

1. Entry criteria

Characteristic clinical or radiologic involvement of a typical organ (eg. pancreas, salivary glands, bile ducts, orbits, kidney, lung, aorta, retroperitoneum, pachymeninges, or thyroid gland [Riedel’s thyroiditis]) OR pathologic evidence of an inflammatory process accompanied by a lymphoplasmacytic infiltrate of uncertain etiology in one of these same organs.

1. Exclusion criteria:

2.1 Clinical

Fever

No objective response to glucocorticoids

- 1. Serologic

Leukopenia and thrombocytopenia with no explanation

Peripheral eosinophilia

Positive antineutrophil cytoplasmic antibody (specifically against proteinase 3 or myeloperoxidase)

Positive SSA/Ro or SSB/La antibody

Positive double-stranded DNA, RNP, or Sm antibody

Other disease-specific autoantibody

Cryoglobulinemia

- 1. Radiologic

Known radiologic findings suspicious for malignancy or infection that have not been sufficiently investigated

Rapid radiologic progression

Long bone abnormalities consistent with Erdheim-Chester disease

Splenomegaly

- 1. Pathologic

Cellular infiltrates suggesting malignancy that have not been sufficiently evaluated

Markers consistent with inflammatory myofibroblastic tumor

Prominent neutrophilic inflammation

Necrotizing vasculitis

Prominent necrosis

Primarily granulomatous inflammation

Pathologic features of macrophage/histiocytic disorder

- 1. Known diagnosis of the following:

Multicentric Castleman’s disease

Crohn’s disease or ulcerative colitis (if only pancreatobiliary disease is present)

Hashimoto thyroiditis (if only the thyroid is affected)

1. If case meets entry criteria and does not meet any exclusion criteria, according to the histopathological and immunostaining characteristics, serum IgG4 concentration and features of different organs involved (including bilateral lacrimal, parotid, sublingual, submandibular glands, chest, pancreas and biliary tree, kidney, retroperitoneum), on the basis of the corresponding criteria given by the guideline point-by-point, categorical assessment or numeric weight were made (Please refer to the Ref10 for details).
2. The cumulative total points ≥20 could confirm the final diagnosis.

**Rules and regulations for IgG4-RD RI scoring (9)**

All the following organs were scored separately according to the Scoring Rules, and the scores of each organs were summed to obtain the final RI score.

Scoring Rules

Scoring refers to manifestations of disease activity present in the last 28 days

Scoring: 0-Normal or resolved

1-Improved but still present

2-New / Recurrence while patient is off treatment or unchanged from the previous visit*

3-Worsened or new disease manifestation despite treatment

*(*Unchanged from previous visit will often refer to disease manifestations that require follow-up imaging to assess accurately.*

*Presence of urgent disease within an organ leads to DOUBLING of that organ system score.)*

Potential sites of disease activity captured using the IgG4-RD Responder Index scoring scheme

| Meninges | Pituitary gland |
| --- | --- |
| Orbital lesion | Lacrimal gland |
| Parotid gland | Submandibular gland |
| Other salivary gland | Mastoiditis/middle ear disease |
| Nasal cavity lesion | Sinusitis |
| Other ear, nose, and throat lesion | Thyroid |
| Lung | Lymph node |
| **Aorta/large blood vessel** | **Heart/pericardium** |
| Retroperitoneal fibrosis | Sclerosing mediastinitis |
| Sclerosing mesenteritis | Pancreas |
| Liver | Bile duct |
| Kidney | Skin |
| Constitutional symptoms (weight loss, fever, fatigue due to IgG4-RD) | Other |

Only two sites of cardiovascular involvement was involved: “Aorta/large blood vessel” and “Heart/pericardium”, so according to our CMR and coronary CE results, myocardial, cardiac function, pericardial and coronary involvement were all included in the “Heart/pericardium” category.

Because the cardiovascular involvement was identified for the first time as a new disease manifestation in all IgG4-RD patients in our cohort, the relevant category could only be assigned a score of 3. That is, assigned a score of 3 in “Heart/pericardium” category whenever a patient had either myocardial, cardiac function, pericardial, or coronary involvement.

**ADDITIONAL FIGURES
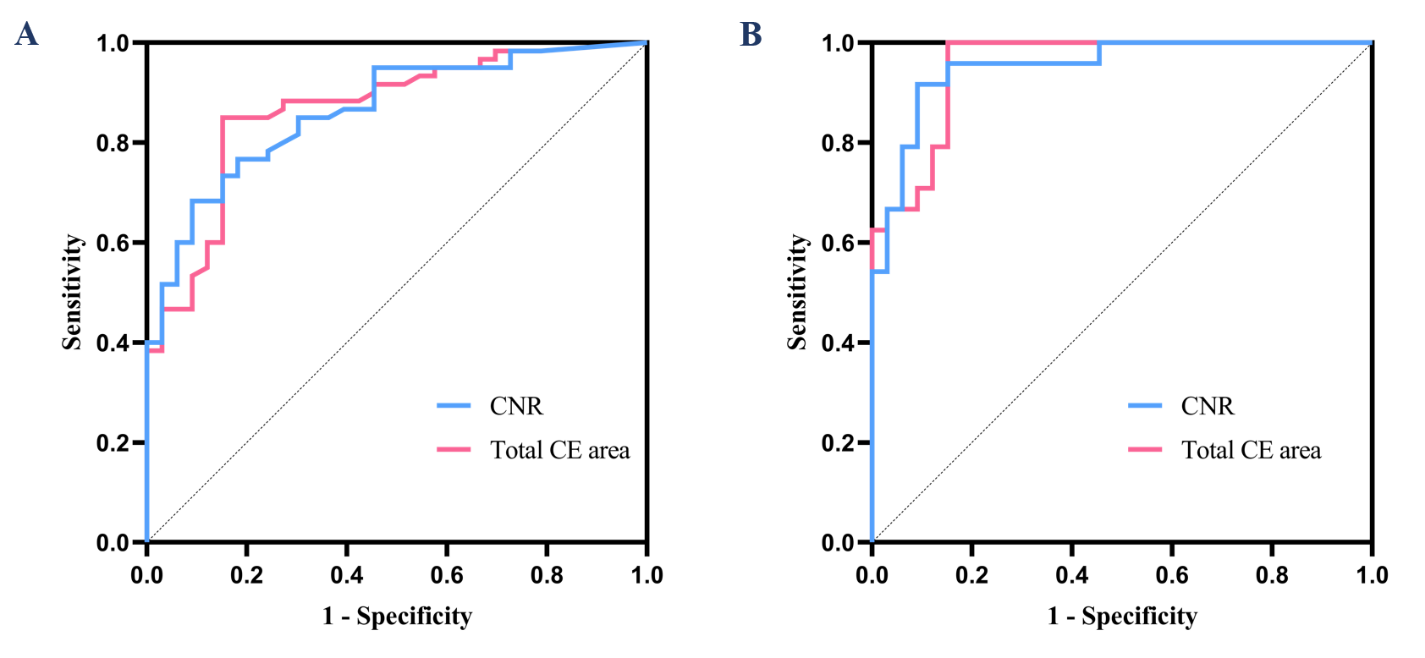
**

**Additional Figure 1. Receiver-operating characteristic curve in evaluating diagnostic efficiency of coronary contrast enhancement in the whole corhort (A) and untreated IgG4-RD patients and controls (B).**

(A). Total CE area: cut-off value=0.99 cm^2^, sensitivity 85%, specificity 85%, area under the curve: 0.87, odds ratio = 3.65, 95% confidence interval: 1.96 to 6.78, *p* < 0.001. CNR: cut-off value=3.85, sensitivity 68%, specificity 91%, area under the curve: 0.86, odds ratio = 2.14, 95% confidence interval: 1.55 to 2.96, *p* < 0.001.

(B). Total CE area: cut-off value=0.99 cm^2^, sensitivity 99%, specificity 85%, area under the curve: 0.95, odds ratio = 5.22, 95% confidence interval: 2.11 to 12.89, *p* < 0.001. CNR: cut-off value=3.85, sensitivity 92%, specificity 91%, area under the curve: 0.95, odds ratio = 3.52, 95% confidence interval: 1.86 to 6.68, *p* < 0.001.

**
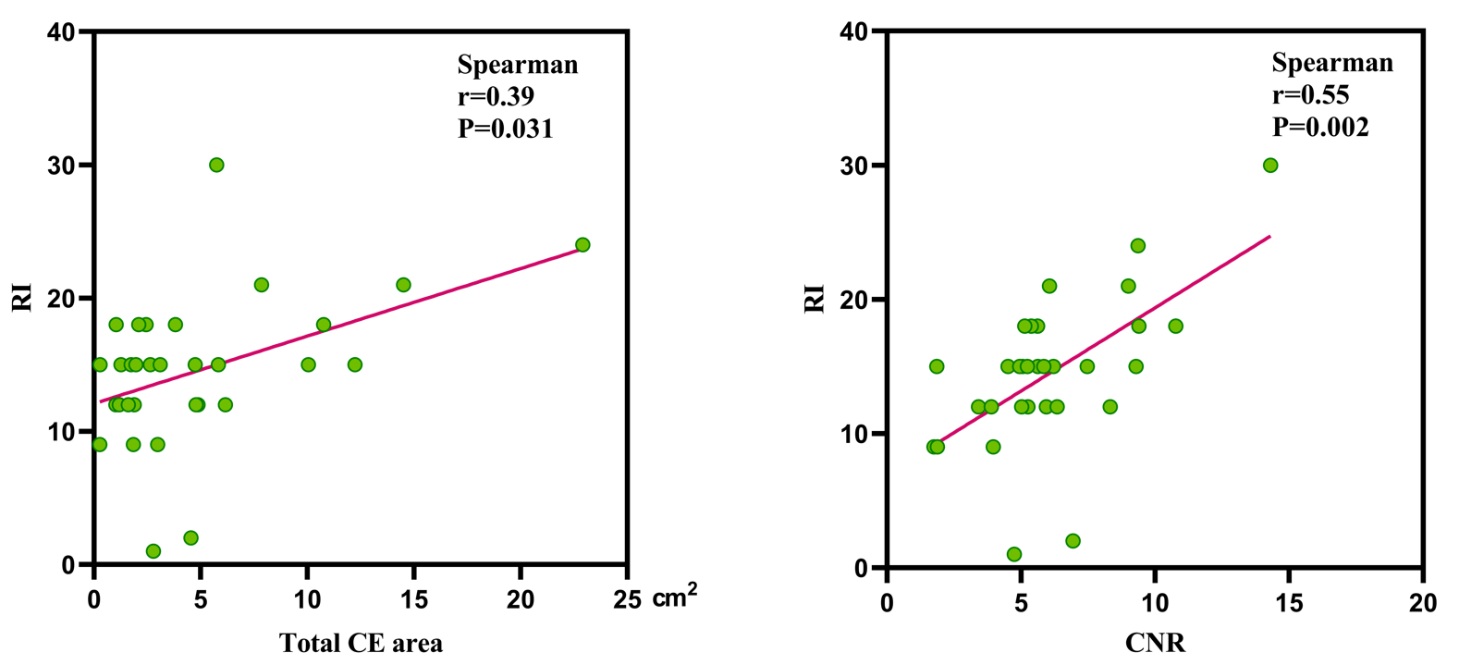
**

**Additional Figure 2. Correlation Analysis between coronary contrast enhancement (CE) and response index (RI) scores in IgG4-related disease (IgG4-RD) patients.** CNR and RI showed moderate positive correlation, while total CE area and RI showed mild positive correlation. Each data point represents the mean CNR and total CE area determined from independent measurements made by the 2 readers.

CNR=contrast to noise ratio.

**ADDITIONAL TABLES**

**Additional Table 1.** **The updated response index (denoted as RI’) with consideration of coronary wall contrast enhancement versus the prior RI** **without consideration of coronary wall contrast enhancement**

|  | RI | | | RI’ | | |
| --- | --- | --- | --- | --- | --- | --- |
|  | Cardiac | Non-cardiac | Total | Cardiac | Non-cardiac | Total |
| 1 | 3 | 27 | 30 | 3 | 27 | 30 |
| 2 | 0 | 15 | 15 | 3 | 15 | 18 |
| 3 | 0 | 18 | 18 | 3 | 18 | 21 |
| 4 | 3 | 15 | 18 | 3 | 15 | 18 |
| 5 | 3 | 9 | 12 | 3 | 9 | 12 |
| 6 | 0 | 2 | 2 | 3 | 2 | 5 |
| 7 | 0 | 18 | 21 | 3 | 18 | 21 |
| 8 | 0 | 15 | 15 | 3 | 15 | 18 |
| 9 | 0 | 15 | 15 | 3 | 15 | 18 |
| 10 | 0 | 9 | 9 | 0 | 9 | 9 |
| 11 | 0 | 1 | 1 | 3 | 1 | 4 |
| 12 | 0 | 12 | 15 | 3 | 12 | 15 |
| 13 | 0 | 15 | 15 | 3 | 15 | 18 |
| 14 | 0 | 21 | 21 | 3 | 21 | 24 |
| 15 | 3 | 12 | 15 | 3 | 12 | 15 |
| 16 | 0 | 12 | 12 | 3 | 12 | 15 |
| 17 | 0 | 18 | 18 | 3 | 18 | 21 |
| 18 | 0 | 24 | 24 | 3 | 24 | 27 |
| 19 | 3 | 12 | 15 | 3 | 12 | 15 |
| 20 | 0 | 9 | 9 | 3 | 9 | 12 |
| 21 | 3 | 9 | 12 | 3 | 9 | 12 |
| 22 | 0 | 12 | 12 | 3 | 12 | 15 |
| 23 | 0 | 18 | 18 | 3 | 18 | 21 |
| 24 | 3 | 12 | 15 | 3 | 12 | 15 |
| 25 | 3 | 12 | 15 | 3 | 12 | 15 |
| 26 | 0 | 12 | 12 | 3 | 12 | 15 |
| 27 | 0 | 9 | 9 | 3 | 9 | 12 |
| 28 | 0 | 15 | 15 | 0 | 15 | 15 |
| 29 | 0 | 15 | 18 | 3 | 15 | 18 |
| 30 | 3 | 9 | 9 | 3 | 9 | 12 |
| 31 | 3 | 18 | 18 | 3 | 18 | 21 |
| Mean ± SD | 1±1 | 14±5 | 15±6 | 3±1 | 14±5 | 16±6 |

Note: The maximum limit for the "heart/pericardium" category, inclusive of but not limited to coronary involvement, is 3 points. Therefore, in some cases, even with the new detection of coronary wall contrast enhancement, there may be no change in RI’ compared to RI scores.

**Additional Table 2. Characteristics of coronary CE in untreated IgG4-RD patients and controls**

|  | IgG4-RD  (n=24) | Controls  (n=33) | P Value |
| --- | --- | --- | --- |
| Coronary artery wall thickening | 10（42） | 0（0） | ＜0.001 |
| Coronary CE | 24（100） | 4（12） | ＜0.001 |
| CE pattern |  |  |  |
| Patchy | 4（17） | 4（100） | 0.227 |
| Generalized | 20（83） | 0（0） | ＜0.001 |
| Total area, cm2 | 4.2(1.9-7.4) | 0.3(0.3-0.9) | ＜0.001 |
| CNR | 6.6±2.7 | 1.9±1.5 | ＜0.001 |
| Values are mean±SD or n (%) or median (interquartile range).  LGE: Late gadolinium enhancement; CE: contrast enhancement;  CNR: contrast to noise ratio; IgG4-RD= IgG4-Related Disease. | | | |

**Additional Table 3. Results of correlation analysis in untreated IgG4-RD patients**

|  | Total CE area  r p Value | CNR  r p Value |
| --- | --- | --- |
| Total CE area |  | 0.83 ＜0.001 |
| CNR | 0.83 ＜0.001 |  |
| IgG | 0.58 0.003 | 0.41 0.054 |
| IgG4 | 0.61 0.001 | 0.42 0.041 |
| C3 | -0.39 0.044 | -0.43 0.042 |
| C4 | -0.18 0.416 | -0.24 0.279 |
| CRP | 0.23 0.298 | -0.07 0.795 |
| ESR | 0.56 0.005 | 0.35 0.105 |
| IgG4-RD RI 0.49 0.015 0.73 ＜0.001 | | |
| Correlations were performed using Pearson or Spearman tests, as appropriate for the type of the data.  IgG4-RD RI= IgG4-Related Disease Responder Index; CRP: C-reactive protein; ESR: erythrocyte sedimentation rate. | | |
